# Adsorptive Capture of Ionic and Non-Ionic Pollutants Using a Versatile Hybrid Amphiphilic-Nanomica

**DOI:** 10.3390/nano11123167

**Published:** 2021-11-23

**Authors:** Fernando Aguado, Rosa Martín-Rodríguez, Carmen Pesquera, Rafael Valiente, Ana C. Perdigón

**Affiliations:** 1 CITIMAC Department, University of Cantabria, Avda. de Los Castros 48, 39005 Santander, Spain; aguadof@unican.es; 2 Nanomedicine Group, IDIVAL, Avda. Cardenal Herrera Oria s/n, 39011 Santander, Spain; rosa.martin@unican.es (R.M.-R.); carmen.pesquera@unican.es (C.P.); rafael.valiente@unican.es (R.V.); 3 QUIPRE Department, University of Cantabria, Avda. de Los Castros 46, 39005 Santander, Spain; 4 Applied Physics Department, University of Cantabria, Avda. de Los Castros 48, 39005 Santander, Spain

**Keywords:** high charge mica, adsorption, calorimetry, decontamination, ionic pollutants, non-ionic pollutants, Eu^3+^ luminescence

## Abstract

A versatile, functional nanomaterial for the removal of ionic and non-ionic pollutants is presented in this work. For that purpose, the high charge mica Na-4-Mica was exchanged with the cationic surfactant (C_16_H_33_NH(CH_3_)_2_)^+^. The intercalation of the tertiary amine in the swellable nano-clay provides the optimal hydrophilic/hydrophobic nature in the bidimensional galleries of the nanomaterial responsible for the dual functionality. The organo-mica, made by functionalization with C_16_H_33_NH_3_^+^, was also synthesized for comparison purposes. Both samples were characterized by X-ray diffraction techniques and transmission electron microscopy. Then, the samples were exposed to a saturated atmosphere of cyclohexylamine for two days, and the adsorption capacity was evaluated by thermogravimetric measurements. Eu^3+^ cations served as a proof of concept for the adsorption of ionic pollutants in an aqueous solution. Optical measurements were used to identify the adsorption mechanism of Eu^3+^ cations, since Eu^3+^ emissions, including the relative intensity of different *f–f* transitions and the luminescence lifetime, can be used as an ideal spectroscopic probe to characterize the local environment. Finally, the stability of the amphiphilic hybrid nanomaterial after the adsorption was also tested.

## 1. Introduction

Organic–inorganic hybrid nanomaterials represent a clever strategy for designing new functional materials that combine the optimal level of hydrophobicity created by an organic molecule or polymer—required for different industrial uses—with the structural properties of an inorganic component, preferentially thermal and mechanical stability [1]. The intercalation of organic species in lamellar solids, specifically in clay platelets, constitutes an important example of organically modified 2D nanocomposites with a great presence in the industrial market [2,3,4]. In particular, organic nano-clays have been proposed as efficient adsorbents to remove organic pollutants, such volatile organic compounds (VOCs) and other non-ionic hydrocarbons (NOCs), from air and water, because of their numerous advantages, mainly their relatively low price, high surface area and mechanical stability, among others [5,6]. High charge micas are a family of layered aluminosilicates with improved adsorption properties and loading capacities up to four times those of low charged aluminosilicates, such as bentonites; and from which a set of organo-clays has been successfully synthesized [7,8,9]. Additionally, those functionalized nanomaterials, made by incorporating long chain alkylammonium cations in the galleries of the clay, have already demonstrated their capacity to capture different NOCs, such as phenol, benzene and toluene, through an adsorptive removal mechanism [10]. In a similar way, successful removals of some surfactants, perfluoroalkyl and pharmaceutical compounds from aqueous solutions by organo-functionalized micas have been also reported [11,12,13]. Additionally, functionalization of those high charge micas with amine and thiol groups allows the capture of ionic pollutants [14,15].

A deep knowledge of the driving forces involved in the intercalation process and the surface chemistry is crucial to fully controlling the synthesis processes and to understand the adsorption mechanism. Several factors, such as the electrostatic attraction between the surfactant polar head group and the nano-clay surface, the amount and configurations of the organic species inside the layers, the attractive van der Waals forces between tails and the level of hydrophobicity/solubility of the surfactant, play fundamental roles in the final properties and applicability of the products [16]. In that way, we have recently described an experimental route to synthesizing nanostructured organo-micas prepared from primary and tertiary C16 amines. Controlled adsorption of the organics leads to tunable hydrophobicity in the interlayer space of the hybrid material. This provides the possibility to choose the optimal interface nature, as is required for a variety of applications, from a fully hydrophobic medium to an amphiphilic quasi-solution [17]. Specifically, an organo-mica with a homogeneous single-phase organic-clay is formed from an exchange reaction with long primary n-alkylammonium cations. In addition, a synthesis route in which the exchange capacity of the high charge mica is not fully satisfied has been proposed for a more amphiphilic interface, which relies on the incorporation of long tertiary amines. For the adequate synthesis of the first type of functionalized mica, the organo-mica, the length of the surfactant is a crucial parameter to assuring quantitative uptake of the surfactant by the attractive van der Waals interactions between the alkyl chains. However, the head group of the surfactant, in terms of size—steric effects—and nature—hydrophobicity—is the fundamental parameter that controls the final product of the second type of mica, the amphiphilic mica. In the latter case, a hydrated, homogeneous inorganic–organic interlayer is synthesized. This hybrid material allows one to combine exchangeable inorganic cations and adsorbent organic species between the solid layers, creating a promising adsorbent material with dual functionality toward both hydrophilic and hydrophobic pollutants for water and air decontamination. Besides, the surfactant molecules are able to swell the interlayer space of the aluminosilicate, taking the layers apart, making the Lewis acidic centers accessible to contaminants in a complementary adsorption mechanism. Despite the promising features exhibited by this functionalized material, its affinity for inorganic and organic species has not been analyzed in detail before.

We present in this work a deep insight into the dual functionality of the amphiphilic mica as a versatile adsorbent for ionic and non-ionic pollutants. For that purpose, the swellable high charge mica Na-4-Mica was exchanged with the tertiary R-N(CH_3_)_2_ amine with alkyl length R = 16, in an acidic medium. When the Na-4-Mica is exchanged with the tertiary ammonium cation [RNH(CH_3_)_2_]^+^, R = 16, the exchange capacity is not fully satisfied, and a homogeneous heteroionic structure is formed with mixed organic/inorganic cations in the same interlayer [17]. The organo-mica, C_16_H_33_NH_3_^+^-mica, was also synthesized via cation exchange reaction. The length of the primary ammonium cation was carefully chosen to assure that van der Waals interactions between alkyl chains would be strong enough to allow a quantitative uptake of the surfactant cations in this sample [17]. This organo-mica has been previously reported to be an improved adsorbent material of hydrophobic VOCs for water decontamination [10]. Then, both functionalized clays were firstly exposed to a saturated atmosphere with cyclohexylamine. Cyclohexylamine is a strong organic base that is used widely as a corrosion inhibitor, and it is toxic at high exposure levels [18]. Under these unfavorable conditions, a comparative analysis of their adsorption capacities for non-ionic compounds was carried out. In a second step, the sample C_16_H_33_NH(CH_3_)_2_^+^-mica was put in contact with a Eu^3+^ water solution as a proof of concept of its capacity to adsorb inorganic contaminant cations. Eu^3+^ cations incorporated in the interlayer space of high charge mica have been recently proposed as an ideal luminescent probe to determine the sorption behavior and cation environment [19]. Moreover, it has to be mentioned that high charge micas have been proposed as ideal materials to capture radioactive waste. In particular, superselectivity and stable immobilization have been described by Komarneni et al., for ^137^Cs and ^226^Ra, through electrostatic bonding at room temperature [20]. It is standard practice to use the appropriate stable lanthanide cation instead its corresponding actinide as the chemical simulator [21,22,23]. The functionalized high charge micas were firstly characterized by X-ray diffraction (XRD) and transmission electron microscopy (TEM). The physisorbed cyclohexylamine was analyzed by thermogravimetric (TG) and mass spectroscopy analyses. The interlayer exchange of Na^+^ with Eu^3+^ in the amphiphilic mica was monitored by luminescence measurements, since Eu^3+^—by it emission, including the relative intensities of different transitions and the luminescence lifetime—can be used as an ideal spectroscopic probe to characterize the local environment of Eu^3+^. For that purpose, a doped Eu^3+^ Mica-4 was also synthesized and characterized by XRD, TG and optical measurements.

## 2. Materials and Methods

### 2.1. Synthesis of High Charge Micas

Na-4-Mica, with four negative charges per unit cell in its structure and ideal chemical formula Na_4_[Mg_6_Si_4_Al_4_O_20_F_4_] · H_2_O, was synthesized following the “NaCl method” described by Park et al. [24]. Stoichiometric amounts of SiO_2_, (from Sigma, purity 99.8%), Al(OH)_3_ (from Riedel-de-Haën, purity 99.7%), MgF_2_ (from Aldrich, purity 99.9%) and twofold the stoichiometric amount of NaCl (from Panreac, purity 99.9%) were well mixed in an agate mortar. Reactants were thermally treated in a Pt crucible at 900 °C for 15 h and left to cool down. After cooling, the solid was washed with deionized water to eliminate the excess of NaCl and dried at room temperature.

### 2.2. Synthesis of the Organo and the Amphiphilic-Mica

The chemical products used for the preparation of the organo-mica and the amphiphilic mica, hexadecylamine (RNH_2_) and dimethylhexadecylamine [RN(CH_3_)_2_] with R = 16, respectively, were obtained from Aldrich Chemical Co. Neutral amines, were firstly converted to the protonated form by adding them in an aqueous solution of 0.1 M HCl, in a molar ratio amine:HCl 1:1, and stirred at 80 °C for 3 h. Then, 1 g of Na-4-Mica was added to the protonated amines and they were left to react for 24 h at 80 °C. A two-fold excess of the clay cation exchange capacity (CEC) of the amines was used in order to favor the cation exchange reaction. Both the organo-clay and the amphiphilic clay were recovered by centrifugation, washed with deionized water and ethanol and dried at room temperature.

### 2.3. Adsorption of Cyclohexylamine and Eu^3+^Cations

The organo-mica and the amphiphilic mica were put in contact with a saturated atmosphere of cyclohexylamine for 48 h. For the adsorption of Eu^3+^, 300 mg of Mica-4 and the amphiphilic sample were dispersed in 50 mL of a Eu(NO_3_)_3_ (REacton 99.9%) water solution 0.01 M, respectively. The process was repeated three more times. Then, the samples were centrifuged and washed with deionized water.

### 2.4. Characterization

XRD patterns were obtained with a Bruker D8 Advance instrument using Cu Kα radiation at 40 kV and 30 mA. Diffractograms were obtained from 1.5° to 70° (2*θ*) at a scanning speed of 0.05 deg·min^−1^ and a counting time of 5 s.

TG analysis was performed on a Setaram Setsys evolution TGA-DTA/DSC model. The sample was heated from room temperature to 800 °C at a heating rate of 10 °C min^−1^ in air. Approximately 20 mg of sample was heated up in an open platinum crucible.

TEM images were obtained on a JEOL JEM 2100 microscope with a CeB_6_ filament. TEM samples were prepared by sonication of the powder in ethanol and evaporating one drop onto a holey carbon film on top of a copper grid.

Steady state luminescence, excitation and lifetime measurements were performed using a FLS920 spectrofluorometer (Edinburgh Instruments) equipped with double-monochromators, a continuous Xe-lamp of 450 W and a pulsed Xe-lamp of 60 W (µF920) for excitation and a Hamamatsu R928 photomultiplier tube (PMT) for detection. All emission spectra were corrected for the system response.

## 3. Results and Discussion

### 3.1. Functionalization of Na-4-Mica

**Figure 1:**
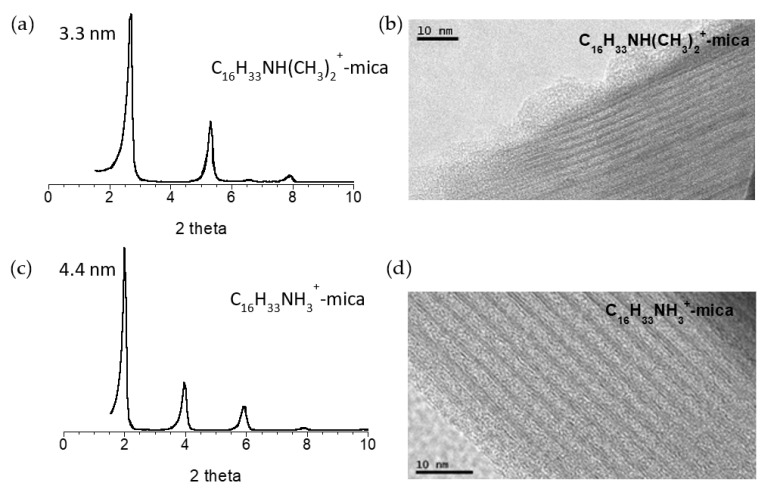
XRD patterns of the amphiphilic mica, C_16_H_33_NH(CH_3_)_2_^+^-mica (**a**), and the organo-mica, C_16_H_33_NH_3_^+^-mica (**c**); and TEM images of the amphiphilic mica (**b**) and the organic-mica (**d**).

Figure 1 includes the diffraction analysis and TEM images of the starting samples; the organo-mica, C_16_H_33_NH_3_^+^-mica; and the amphiphilic mica, C_16_H_33_NH(CH_3_)_2_^+^-mica. The XRD diagram of the organo-mica shows a principal reflection attributed to the (001) basal reflection with an associated *d* spacing value of 4.4 nm. The high interlayer space has been previously associated with a paraffin bilayer structure of the surfactant with double all-trans conformation, with the hydrophobic tails pointing toward the interlayer space tilted 58.2° away from the clay surface [7,17]. The existence of other (001) reflections in the diffraction pattern, and the regularly ordered layered structure that can be observed from the TEM image in the figure, confirm the homogeneous distribution and dense packaging of the surfactant in the galleries of the clay. As a consequence, a hydrophobic/hydrophilic hybrid material, made of alternating hydrophobic galleries fully occupied by surfactant cations and hydrophilic clay layers, was generated for the full displacement of the interlayer sodium cations by the organic species.

In addition, due to the attraction between the organic tails, a quantitative uptake of the surfactant and even additional adsorption have been described for surfactants with long alkyl chains, since the van der Waals forces are proportional to the number of CH_2_ groups (1-1,5 kJ per CH_2_ group) [25]. For the other sample, the amphiphilic mica, the XRD diagram exhibited a (001) reflection at 2.7° 2θ with a basal space of 3.3 nm, compatible with a paraffin bilayer arrangement of the dimetilhexadecylammonium surfactant on the galleries of the silicate, with a tilting angle of 32.2°. This tilting angle of 32.2° together with the displacement of the (001) reflection up to 3.3 nm suggest partial replacement of the hydrated sodium cations by the organics. It was confirmed by TG measurements. Additionally, the formation of a heterostructure or a segregation arrangement can be discarded by the absence of a second basal reflection family corresponding to hydrated sodium in the interlayer [17]. The presence of organic cations on the C_16_H_33_NH(CH_3_)_2_^+^-mica and C_16_H_33_NH_3_^+^-mica samples was also corroborated by infrared measurements, and the results are included as Supplementary Materials.

### 3.2. Adsorption of Cyclohexylamine

**Figure 2:**
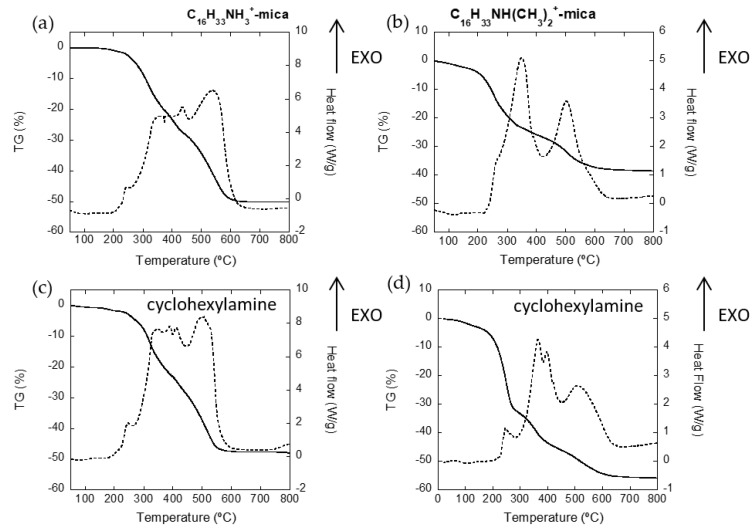
TG (solid line) and DSC (dashed line) plots for C_16_H_33_NH_3_^+^-mica (**a**), C_16_H_33_NH(CH_3_)_2_^+^-mica (**b**) and the samples after cyclohexylamine adsorption (**c**,**d**).

Firstly, cyclohexylamine was used as the model for an organic contaminant, and its uptake by the adsorbent materials was estimated using TG measurements. Figure 2 includes the TG-DSC curves of the hybrid materials before and after the adsorption of cyclohexylamine.

Three regions can be identified in the mass loss curve of the organic functionalized micas [26,27,28]. Up to 170 °C, the first mass loss step, associated with an endothermic peak in the heat flow curve, relates to desorption of water from the clay mineral and dehydration of the interlayer cation. For the pure organo-mica, there was not mass loss in that region according to the structure introduced above. However, the mass loss in the amphiphilic mica was about 2.2%, corroborating the presence of inorganic cations in the interlayer space. In the second region, between 170 and 500 °C, the mass loss was associated with the thermal oxidation of the organics in the interlayer space of the clay, and it was also accompanied by one or more exothermic peaks in heat flow. The combustion process could be prolonged with higher temperatures, depending on the nature of the organic matter, the amount of surfactant adsorbed and the oxygen availability.

While the potential use of this organo-mica as an effective adsorbent of non-ionic pollutants has been previously probed in aqueous solutions, the adsorption capacity for non-ionic contaminants in air has not been tested before. The efficiency of the samples in cyclohexylamine uptake after being exposed to a saturated atmosphere for two days was comparatively studied, and the respective amounts of cyclohexylamine adsorbed were evaluated by TG measurements. The amount of adsorbed cyclohexylamine is attributed to the extra mass loss shown in the second region of the thermogram. Under these unfavorable conditions compared with an aqueous medium, TG measurements show that cyclohexylamine was not adsorbed in the organo-mica, probably due to the large number of surfactant molecules closely packed in the interlayer space. In this sample, the surfactant was adsorbed in higher amounts than its cation exchange capacity suggests, so the extra organic molecules could have been in the organo-layer saturating the adsorption centers. On the contrary, cyclohexylamine was adsorbed in the amphiphilic sample. The amount of adsorbed cyclohexylamine was estimated to be 16% of the sample mass.

TG experiments of the initial Mica-4 exchanged with C_16_H_33_NH(CH_3_)_2_^+^ ions showed a ratio of replacement of 50% of the interlayer Na^+^ (~38% mass loss). The hybrid material was then organized in a regularly intercalated layered phase, with surfactant cations and sodium cations in an associative distribution, forming hydrophobic and hydrophilic independent clusters in the interlayer space of the aluminosilicate [17]. The composition of the interlayer agrees with the results extrapolated from the TEM image and the XRD diagram included in Figure 1. In the amphiphilic mica, a homogenous distribution of the organic and inorganic clusters along the interlayer provides the sample with the adequate level of hydrophobicity to facilitate the incorporation of cyclohexylamine in the functionalized material. Additionally, chemical adsorption of the primary amine onto the Lewis acidic centers, exposed on the silicate surface, can help in the adsorption process. Under this premise, the TG measurements showed satisfactory preliminary results for the adsorption of non-ionic or hydrophobic VOCs when using cyclohexylamine as a tester molecule.

**Figure 3:**
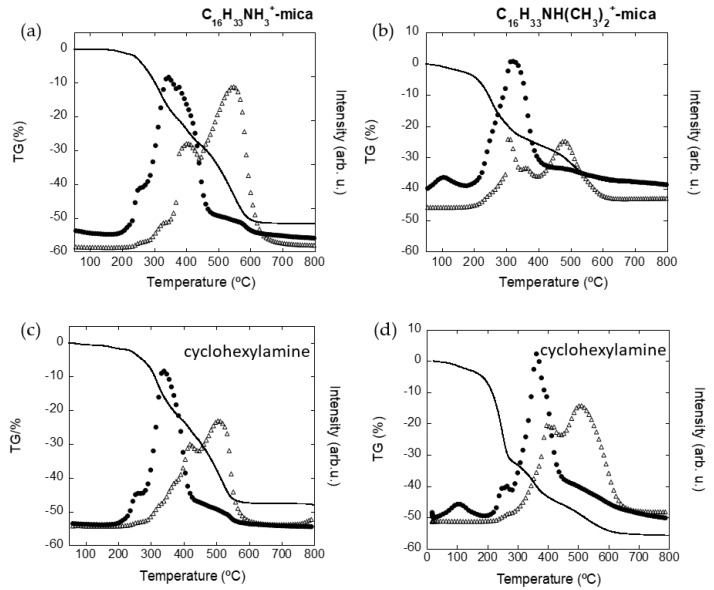
TG (solid line), MS H_2_O curves (black dots) and MS CO_2_ curves (triangles) of C_16_H_33_NH_3_^+^-mica (**a**), C_16_H_33_NH(CH_3_)_2_^+^-mica (**b**) and the samples after cyclohexylamine adsorption (**c**,**d**).

TG-DSC measurements were also monitored by acquiring the CO_2_ and H_2_O signals of the evolved gas from the thermal treatment using mass spectrometry. The results are included in Figure 3. Below 170 °C, the mass loss observed in the thermogram of the amphiphilic mica, associated with the dehydration process of the inorganic interlayer cations, was confirmed by a maximum in the water signal at 100 °C (Figure 3b,d). However, this maximum is absent for the organic sample in the water signal (Figure 3a,c). As result of the combustion of organic matter in an air atmosphere at temperatures below 450 °C, the mass loss process in all the samples reached a maximum alongside maxima for both water and CO_2_ signals. This process was prolonged up to a third region where the residual charcoal was fully oxidized above 500 °C, evidenced by a maximum in the CO_2_ curve [29]. Thus, mixed ion clays, combining both potentially exchangeable adsorbent organics species and inorganic cations, represent significant new forms of decontaminant for both non-ionic or hydrophobic VOCs and cationic inorganic ions, such as heavy metals.

### 3.3. Adsorption of Eu^3+^Cations

Once the capacity for retention of cyclohexylamine was demonstrated in the amphiphilic sample, the adsorption spectrum of inorganic contaminants was analyzed using Eu^3+^ as a cationic model. The optical properties of the amphiphilic clay upon contact with a solution of europium nitrate have been studied in detail. Excitation and luminescence measurements of Eu^3+^ were previously used, considering Eu^3+^ as a probe, to explore the adsorption mechanism of cationic species in high charge micas [30].

Firstly, the structural and optical properties of a doped Eu^3+^ Mica-4 were analyzed for comparative purposes. Importantly, the structure of high charge micas presents some particular advantages for use as luminescent sensors to explore the adsorption mechanisms of inorganic cations: (1) The absence of undesirable impurities such as iron in the structure that can cause luminescence quenching. (2) High charge micas are fluorinated clays with poorly hydrated cations in the interlayer space. It is well known that hydroxyl groups cause luminescence deactivation through nonradioactive processes. (3) Interlayer cations are homogenously distributed along the surface of the aluminosilicate, preventing aggregation of lanthanide ions.

**Figure 4:**
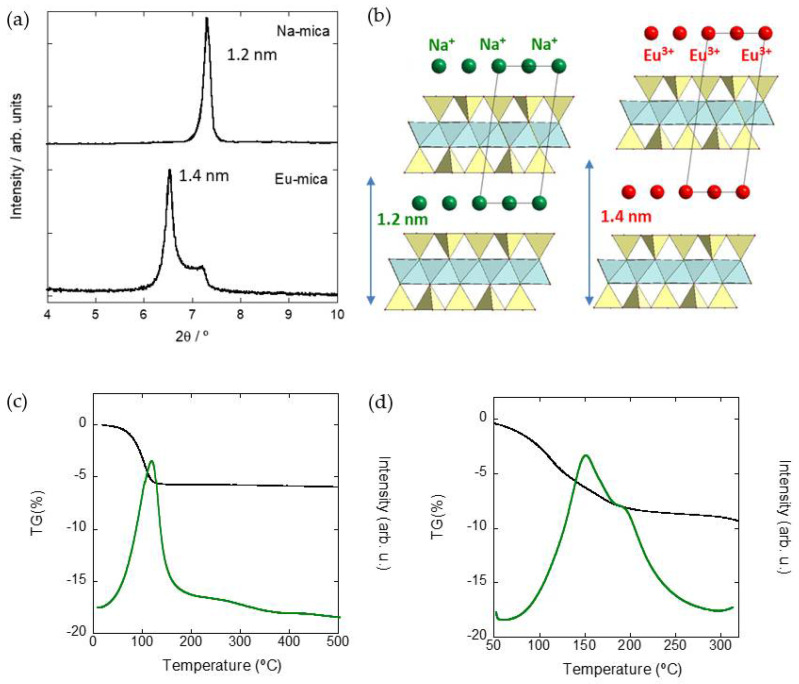
XRD pattern of Na-Mica-4 and Eu^3+^-doped Mica-4 (**a**) and schematic representation of Mica-4 with Na^+^ cations and Eu^3+^ cations in the interlayer space, respectively (**b**). TG (solid line), MS H_2_O curve (green) of Na^+^-Mica-4 (**c**) and Eu^3+^-Mica-4 (**d**).

Figure 4 includes the XRD patterns of a Na-Mica-4 and a Eu^3+^-doped Mica-4 sample and a schematic representation of the unit cell with sodium and europium cations in the interlayer space, respectively. The (001) basal reflection of the as-synthesized mica, situated at 2*θ* ~7.4°, corresponds to a spacing value of *d* = 1.2 nm, and it is associated with Na^+^ cations accommodated in the hexagonal cavities of the tetrahedral sheet, and a pseudo-monolayer of water between the silicate layers, according to the literature [31,32]. The diagram of the Eu^3+^-doped sample exhibits two (001) basal reflections, the most intense situated at 2*θ* ~6.5° and a shoulder at 2*θ* ~7.4°. Since the basal reflection situated at 2*θ* = 6.5° (basal distance = 1.4 nm) is associated with hydrated trivalent europium cations in the inner sphere complex located in the ditrigonal holes of the aluminosilicate, the basal reflection at 7.4° (distance = 1.2 nm) is attributed to hydrated Na^+^ cations in the interlayer space of the mica type clay [19,33]. Attending to the relative intensities of the peaks, an almost complete interlayer cation exchange of Eu^3+^ by Na^+^ can be deduced from the XRD diagram, where a predominance of Eu^3+^-exchanged Mica-4 coexists with a minority of Na^+-^exchanged Mica-4. Figure 4 also shows the TG analysis of Mica-4 before (c) and after (d) the exchange of interlayer Na^+^ with Eu^3+^. The TG measurements were also followed by measuring the water vapor evolution signal with temperature through mass spectrometry (in green). The mass loss curve of swelling phyllosilicates is characterized by one mass loss step below 250 °C corresponding to desorption of water from the clay’s surface and dehydration of the interlayer cation. Mica-4 has a mass loss step of ca. 6% associated with a maximum in the water vapor curve at ~100 °C. The number of water molecules in the coordination sphere for each Na^+^ cations has been calculated to be 0.7 from the 6% water loss, in agreement with previous reports. The sample exchanged with Eu^3+^ cations showed more than one mass loss step below 250 °C, suggesting the existence of water molecules attached to the silicate through bonds with various levels of strength.

The associated water vapor curve presents two maxima at ~130 and ~180 °C. The mass loss of the Eu^3+^-exchanged Mica-4 was ~9%, which corresponds to 3.8 water molecules per cation. It has to be mentioned that the whole amount of water was considered to identify the coordination sphere of Eu^3+^ in the aluminosilicate from TG measurements. In both samples, the interlayer cation adopted an inner-sphere conformation, since the existing water molecules were not numerous enough to fully address the coordination sphere of the interlayer cation. Thus, the coordination sphere would have been completed with the basal oxygens of the tetrahedral sheet.

**Figure 5:**
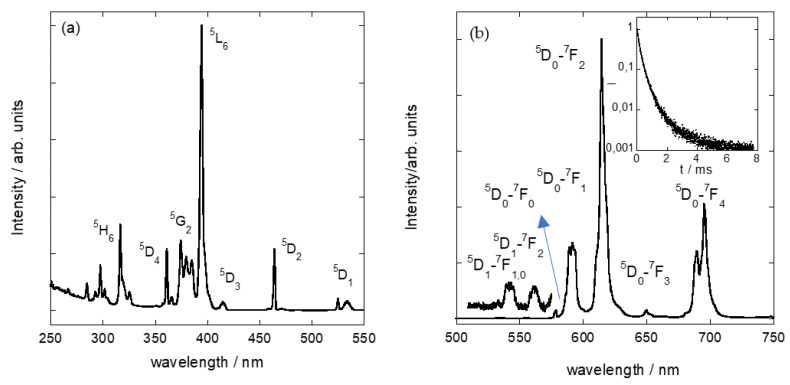
Excitation spectrum of Eu^3+^-doped Mica-4 recording Eu^3+^ at 610 nm (**a**) and the emission spectrum of Eu^3+^-doped Mica-4 upon excitation at 394 nm (**b**). The inset shows the time dependence of the Eu^3+^ emission intensity on a semilog scale.

**Figure 6:**
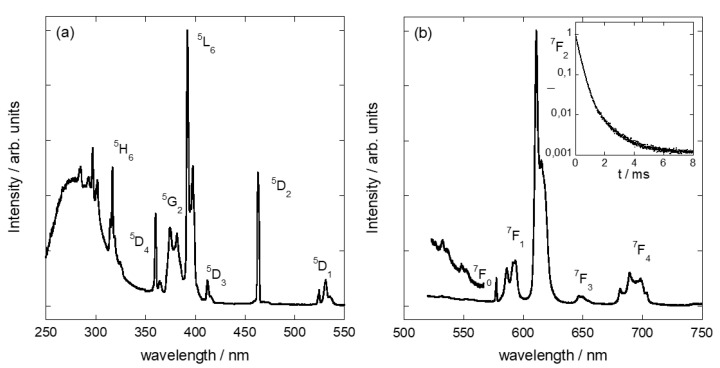
Excitation spectrum of Eu^3+^ adsorbed on C_16_H_33_NH(CH_3_)_2_^+^-mica recording Eu^3+^ at 610 nm (**a**), and emission spectrum of Eu^3+^ adsorbed on C_16_H_33_NH(CH_3_)_2_^+^-mica upon excitation at 394 nm (**b**). The inset shows the time dependence of the Eu^3+^ emission intensity on a logarithmic scale.

Sharp-line Eu^3+^ luminescence has been successfully used to study the adsorption mechanisms of inorganic cations in different crystalline materials [34,35,36]. However, the emission bands from Eu^3+^ adsorbed on other silicates are broad and not well resolved, giving limited information about the closed environments of the luminescent cations in the host materials. Most recently, the optical properties of Eu^3+^ in contact with a high charge mica have been presented as an ideal tool to monitor the cation environment within the clay [14]. Figure 5 includes the excitation (a) and luminescence (b) spectra of Eu^3+^ incorporated in the interlayer of a Mica-4 sample. The excitation spectrum, recorded at 610 nm, consists of a set of narrow peaks associated with *f–f* Eu^3+^ transitions from the ^7^F_0_ ground state to the different excited states marked in the Figure 5. The emission spectrum of Eu^3+^-doped Mica-4 was recorded upon excitation at 394 nm. Only the luminescence from the ^5^D_0_ Eu^3+^ excited state is usually observed in clay minerals; however, the emission spectrum of the Eu^3+^-doped Mica-4 is composed of sharp emission bands from both the ^5^D_1_ and ^5^D_0_ excited states to the ^7^F*_J_* (*J* = 0–4) low-lying multiplets. The presence of the green Eu^3+^ emission from the ^5^D_1_ excited state is associated with Eu^3+^ cations in the interlayer space of the aluminosilicate, in agreement with the XRD results. The inset in Figure 6 shows the temporal evolution of the ^5^D_0_ Eu^3+^ emission on a semilog scale. Clearly, non-single exponential behavior is shown. As stated before, the ^5^D_0_ → ^7^F*_J_* (*J* = 0, 3) transitions are highly sensitive to the local environment of the Eu^3+^ [36]. Thus, analysis of the Eu^3+^ optical properties, namely, luminescence intensity and lifetime, provides valuable information regarding the adsorption process of an inorganic cation.

Figure 6 shows the excitation and emission spectra of Eu^3+^ incorporated in the amphiphilic mica. The observed peaks are assigned to the same transitions as in Figure 5. The temporal evolution of Eu^3+^ luminescence is also presented in the inset of Figure 6b. Clearly, the decay curve is identical to the one observed for Eu^3+^ in the interlayer of Mica-4 (Figure 5b). This represents the first evidence of Eu^3+^’s incorporation in the interlayer of the amphiphilic sample. Although its intensity is low (note the scale factor), the observation of the green Eu^3+^ emission from the ^5^D_1_ level is also consistent with the cation’s incorporation in the amphiphilic mica interlayer. The ^5^D_0_ → ^7^F_0_ transition is very sensitive to the environment; thus, the fact that it is slightly blue-shifted, narrower and more intense for the amphiphilic mica, compared to the Mica-4 sample, is the result of small differences in Eu^3+^–ligand angles and distances. The intensity ratio of the ^5^D_0_ → ^7^F_2_ and ^5^D_0_ → ^7^F_1_ transitions, *R*, is usually used as an indication of the Eu^3+^ site’s asymmetry. The fact that we found *R* to be two-fold larger for the amphiphilic mica than the high charge mica evidences stronger distortion of the Eu^3+^ coordination complex in the former.

**Figure 7:**
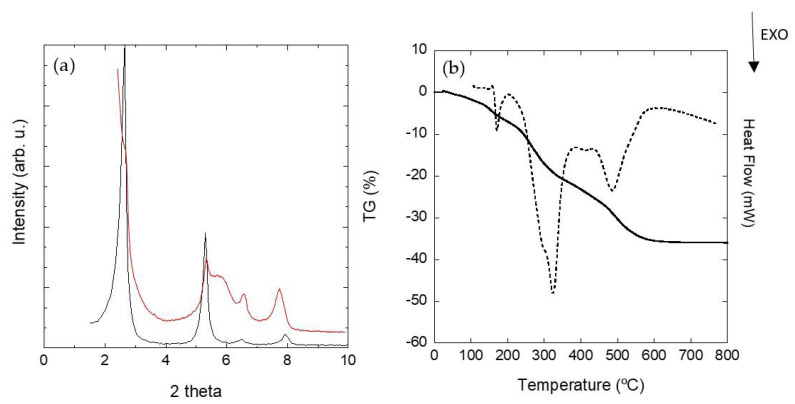
XRD patterns of the amphiphilic mica, C_16_H_33_NH(CH_3_)_2_^+^-mica, before (black) and after the Eu(NO_3_)_3_ treatment (red) (**a**); TG (solid line) and DSC (dashed line) plots for on C_16_H_33_NH(CH_3_)_2_^+^-mica after Eu^3+^ cation adsorption (**b**).

Finally, to assure the stability of the functionalized Mica-4 after the adsorption of Eu^3+^cations, the sample was analyzed through XRD and TG techniques. Figure 7b includes the mass loss curve and DSC analysis from room temperature up to 800 °C. The mass loss in the first step—below 170 °C—which is attributed to the loss of water molecules adsorbed in the solid and preferentially from the hydration shell of the interlayer cations, was ~3.4%. The mass loss was accompanied by an endothermic peak in the DSC curve. This value is slightly superior to its equivalent step observed in the amphiphilic sample, C_16_H_33_NH(CH_3_)_2_^+^-mica.

The increment in the amount of water observed in this region can be explained by the incorporation of Eu^3+^ in the solid, as it was suggested by luminescent measurements.

As it was mentioned before, the mass loss in the second region, between 170 and 500 °C, was the result of the combustion process of the organics in the interlayer space of the clay under an oxidative atmosphere, and it was also accompanied by one or more exothermic peaks in the heat flow curve. The mass loss in this second region, after the adsorption of the Eu^3+^ cations, was still the 35% of the sample mass, similar to the 38% described for the starting functionalized mica. This fact proves the stability of the sample when it is dispersed in an aqueous media, and it is indicative of the ability of the amphiphilic mica to be used as adsorbent for inorganic cations. This adsorption probably occurs in the interlayer space of the sample through a cation exchange mechanism by substituting hydrated sodium cations with Eu^3+^. Figure 7a includes the XRD patterns of the amphiphilic mica before (black) and after the treatment with the Eu(NO_3_)_3_ water solution (red). No change was observed in the positions of the (001) basal reflections relative to the alkylammonium cations in the interlayer space. The basal distance was *d* = 3.3 nm for both samples, compatible with the paraffin bilayer arrangement of the dimetilhexadecylammonium surfactant on the galleries of the silicate, with a tilting angle of 32.2°, previously described for the amphiphilic sample. A broad contribution associated with the basal reflection (002) was observed after the Eu^3+^ uptake. Besides, the clusters of surfactant molecules situated in the bidimensional galleries of the aluminosilicate remained unchanged.

## 4. Conclusions

We presented in this paper a versatile hybrid material as adsorbent of both ionic and non-ionic pollutants. This dual functionality comes from a combined hydrophobic/hydrophilic interlayer after the functionalization of the high charge mica, Na-4-Mica, with the tertiary (R-NH(CH_3_)_2_)^+^ amine, the alkyl length being 16 carbons. The ability of the material to absorb non-ionic compounds was tested using cyclohexylamine, a strong base, which is toxic at high exposure levels. From TG measurements, an adsorption capacity of 0.2 g of cyclohexylamine per gram of hybrid material was calculated for the amphiphilic mica, under a saturated atmosphere of cyclohexylamine. Under these experimental conditions, this hybrid material demonstrated better adsorption ability than its homologue, the organo-mica. The amphiphilic sample was then exposed to a Eu^3+^ aqueous solution as a proof of concept of the ability of the adsorbent to incorporate harmful inorganic cations in its structure. Complementarily to XRD, optical measurements of Eu^3+^ served to identify the adsorption mechanism of Eu^3+^ cations. Specifically, upon comparison with the optical properties of Eu^3+^ in the interlayer of Mica-4, both the ^5^D_0_ Eu^3+^ lifetime and the presence of green ^5^D_1_ emission corroborated the presence of Eu^3+^ in the amphiphilic clay interlayer. Finally, the stability of the hybrid material was maintained through and after the adsorption process.

## Data Availability

All datas are available upon reasonable request.

## Supplementary Materials

The following are available online at https://www.mdpi.com/article/10.3390/nano11123167/s1, Figure S1: Infrared spectra of the amphiphilic-mica, C_16_H_33_NH(CH_3_)_2_^+^-mica (red) and the organo-mica, C_16_H_33_NH_3_^+^-mica (black).
