# Supplementary material for: Adsorptive Capture of Ionic and Non-Ionic Pollutants Using a Versatile Hybrid Amphiphilic-Nanomica
Source: Nanomaterials (Basel). 2021 Nov 23;11(12):3167. doi: 10.3390/nano11123167 (PMC8708402; doi:10.3390/nano11123167)
Supplement: Supplementary file 1 [file nanomaterials-11-03167-s001.zip › nanomaterials-1458512-supplementary.pdf]

Supplementary Material

Adsorptive Capture of Ionic and Non-Ionic Pollutants Using a Versatile Hybrid Amphiphilic-Nanomica

Fernando Aguado [1,2], Rosa Martín-Rodríguez [2,3], Carmen Pesquera [2,3], Rafael Valiente [2,4] and Ana C. Perdigón [2,3,*]

[1] CITIMAC Department, University of Cantabria, Avda. de Los Castros, 48, 39005 Santander, Spain; aguadof@unican.es

[2] Nanomedicine Group, IDIVAL, Avda. Cardenal Herrera Oria s/n, 39011 Santander, Spain; rosa.martin@unican.es (R.M.-R.); carmen.pesquera@unican.es (C.P.); rafael.valiente@unican.es (R.V.)

[3] QUIPRE Department, University of Cantabria, Avda. de Los Castros, 46, 39005 Santander, Spain.

[4] Applied Physics Department, University of Cantabria, Avda. de Los Castros, 48, 39005 Santander, Spain.

* Correspondence: perdigonac@unican.es; Tel.: +34–94–2201–592

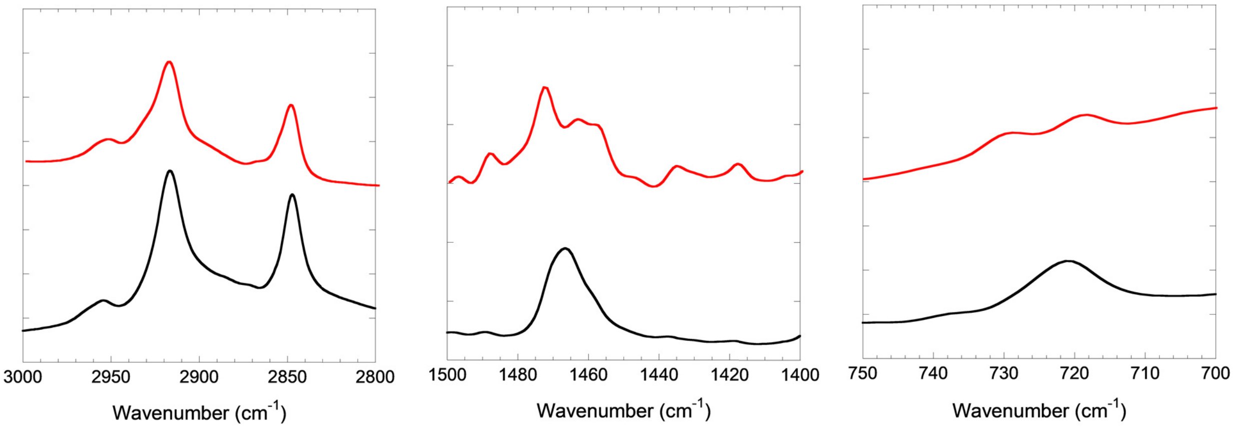

Figure S1. Infrared spectra of the amphiphilic-mica, $C_{16}H_{33}NH(CH_3)_2^+$-mica (red) and the organo-mica, $C_{16}H_{33}NH_3^+$-mica (black).

Figure S1 includes three vibration regions of the FTIR spectra for both samples. Infrared spectra were recorded on a Jasco LE4200 spectrophotometer. The spectra were obtained by accumulation of 264 scans at 4 cm^{-1} resolution, recorded from 4000 cm^{-1} to 400 cm^{-1}. In the first region, from 3000 cm^{-1} to 2800 cm^{-1}, both, the antisymmetric and symmetric CH_2 stretching modes, situated at 2916 cm^{-1} and 2847 cm^{-1}, respectively, as well as a band at 2952 cm^{-1}, attributed to the asymmetric stretching mode of CH_3, can be identified. In the second and third vibrational regions, the presence of CH_2 scissoring modes, between 1480 cm^{-1} and 1440 cm^{-1}, and CH_2 rocking modes, between 750 cm^{-1} and 700 cm^{-1}, also confirm the presence of the organic cations in both samples.
